# Human epicardial adipose tissue expresses a pathogenic profile of adipocytokines in patients with cardiovascular disease

**DOI:** 10.1186/1475-2840-5-1

**Published:** 2006-01-13

**Authors:** Adam R Baker, Nancy F da Silva, David W Quinn, Alison L Harte, Domenico Pagano, Robert S Bonser, Sudhesh Kumar, Philip G McTernan

**Affiliations:** 1 Unit for Diabetes and Metabolism, Warwick Medical School, University of Warwick, Clinical Sciences Research Institute, UHCW Campus, Clifford Bridge Road, Coventry, CV2 2DX, UK; 2 Department of Cardiothoracic Surgery, Queen Elizabeth Hospital, B15 2TH, UK

## Abstract

**Introduction:**

Inflammation contributes to cardiovascular disease and is exacerbated with increased adiposity, particularly omental adiposity; however, the role of epicardial fat is poorly understood.

**Methods:**

For these studies the expression of inflammatory markers was assessed in epicardial fat biopsies from coronary artery bypass grafting (CABG) patients using quantitative RT-PCR. Further, the effects of chronic medications, including statins, as well as peri-operative glucose, insulin and potassium infusion, on gene expression were also assessed. Circulating resistin, CRP, adiponectin and leptin levels were determined to assess inflammation.

**Results:**

The expression of adiponectin, resistin and other adipocytokine mRNAs were comparable to that in omental fat. Epicardial CD45 expression was significantly higher than control depots (p < 0.01) indicating significant infiltration of macrophages. Statin treated patients showed significantly lower epicardial expression of IL-6 mRNA, in comparison with the control abdominal depots (p < 0.001). The serum profile of CABG patients showed significantly higher levels of both CRP (control: 1.28 ± 1.57 μg/mL vs CABG: 9.11 ± 15.7 μg/mL; p < 0.001) and resistin (control: 10.53 ± 0.81 ng/mL vs CABG: 16.8 ± 1.69 ng/mL; p < 0.01) and significantly lower levels of adiponectin (control: 29.1 ± 14.8 μg/mL vs CABG: 11.9 ± 6.0 μg/mL; p < 0.05) when compared to BMI matched controls.

**Conclusion:**

Epicardial and omental fat exhibit a broadly comparable pathogenic mRNA profile, this may arise in part from macrophage infiltration into the epicardial fat. This study highlights that chronic inflammation occurs locally as well as systemically potentially contributing further to the pathogenesis of coronary artery disease.

## Introduction

Cardiovascular disease (CVD) and its sequelae are the leading cause of premature death, leading not only to significantly increased mortality rates but also to high levels of morbidity [1]. Whilst the causes of CVD are complex there is increasing evidence suggesting an integral role for inflammation in CVD pathogenesis, with recent research examining therapeutics targeting this aspect of the disease [2,3]. However, the cause of inflammation and its link with CVD is still poorly understood, particularly in humans, due to the difficulty in studying the relevant human tissues.

Previous studies have highlighted the potential importance of adipose tissue in relation to inflammatory burden in CVD, describing the expression and secretion of both pro-inflammatory and protective factors, collectively termed adipocytokines [4]. These factors include tumour necrosis factor alpha (TNF-α), a pluripotent cytokine that is a key mediator of the acute phase response that also affects non-esterified fatty acid (NEFA) metabolism, as well as myocardial contractility [5]. Resistin, a recently identified adipocytokine, has been proposed as a potential link between obesity and inflammation and has been linked to CVD risk [6,7]. Adiponectin exhibits both insulin sensitising, anti-inflammatory and anti-atherogenic properties with serum levels reduced in both type 2 diabetes mellitus (T2DM) and coronary artery disease (CAD) [8,9]. Kawanami *et al *have described directly reciprocal effects of resistin and adiponectin with regard to inflammation in vascular endothelial cells [10]. Adipose tissue also produces further pathogenic adipocytokines including plasminogen activator inhibitor-1 (PAI-1) and angiotensin II (ANG II), the active metabolite of angiotensinogen (AGT), both important in the fibrinolytic and thrombotic pathways [11,12]. Adipose tissue increases the production of these pathogenic adipocytokines in obesity and it is hypothesised that macrophage recruitment into adipose tissue may contribute to this pathogenic response [13,14].

Studies have further established that adipose tissue distribution has significant impact on disease risk with central abdominal fat increasing both CVD and T2DM risk compared with gluteo-femoral fat [15,16]. Such differences in risk may be attributable to the depot specific differences in the expression and secretion of adipocytokines [17,18]. However, whilst many investigations have elucidated the relative pathogenic risk of abdominal and gluteo-femoral adipose tissue, to date, few studies have investigated the adipocytokine profile of epicardial adipose tissue. This depot, situated predominantly on the right-ventricular free wall and the left-ventricular apex [19], has been shown to have a high capacity for non-esterified fatty acid (NEFA) release and is proposed as a source of this preferred metabolite for the myocardium [20]. Whether adipocytokines are also secreted directly into the cardiac tissue is yet to be established and therefore the potential paracrine effect of epicardial adipocytokines on myocardial metabolism and their role in the pathogenesis of CAD is as yet unknown. However, the lack of any fascia between the adipocytes and the myocardial layer does suggest that factors secreted by the adipocytes would readily interact with the adjacent cardiomyocytes. Clinical studies have noted a strong correlation between the fat mass of epicardial adipose tissue, central abdominal fat and the associated risk of T2DM and CVD [21]. Studies by Mazurek and co-workers comparing expression of pathogenic factors between epicardial and subcutaneous fat from the leg in patients with CAD undergoing coronary artery bypass grafting (CABG), also highlight the potential importance of the inflammatory response of epicardial tissue [22].

During the CABG operation, systemic metabolic substrate infusions can be administered providing myocardial protection and promotion of post-operative myocardial function. This includes the glucose, insulin and potassium (GIK) infusion. Both insulin and glucose can mediate changes in adipocytokine expression; therefore, due to the epicardial fat's proximity to the myocardium, there may be significant paracrine action upon myocardial metabolism through changes in adipocytokine secretion by this depot. Consequently the aims of this present study were to: 1) characterise the expression profile of adipocytokines in epicardial fat from patients undergoing CABG compared with adipose tissue from central and thigh fat from those without CAD; 2) analyse serum levels of pro- and anti-inflammatory cytokines in patients undergoing CABG and case controls and finally; 3) assess the potential effect of drug treatments and GIK infusion on epicardial mRNA adipocytokine expression.

## Materials and methods

### Subjects

#### Tissue collection from CAD subjects

Human epicardial adipose tissue (n = 46) was collected from CAD patients undergoing CABG (age: 53.8 ± 4.9 yr; BMI: 27.5 ± 3.3 kg/m^2^), 10 of which were previously undiagnosed and untreated type 2 diabetics. Epicardial biopsies were taken approximately 1 hour post-anaesthetic, with 24 randomised CAD patients receiving peri-operative infusions of GIK (500 mL 40% glucose, 40 mmol/L KCl, 35 iu insulin (Actrapid, Eli Lilly, UK) at 0.75 mL/kg/hr). All tissue was flash frozen immediately on removal, prior to RNA extraction. During CABG sections of reversed sapheno-femoral vein(s) are grafted into the aorto-coronary position so bypassing significantly stenosed atherosclerotic segments of the epicardial coronary arteries. All biopsies were collected in accordance with guidelines of the South Birmingham ethics committee and informed consent was obtained from all patients. Patients were on a range of medications as outlined in Table 1.

**Table 1:** CAD Patient Medications

Medications	% of total subjects (n = 46) receiving treatment
Statin	81
Aspirin	93
β-Blocker	75
ACEI	38
Ca^2+ ^Channel Blockers	51
Nicorandil	34

Chronic treatments received by the CABG patient cohort

#### Tissue collection from non-CAD subjects

Abdominal subcutaneous (n = 30), omental (n = 14) and thigh (n = 13) adipose tissue was also collected from patients without CAD or T2DM (age: 51.0 ± 7.5 yrs; BMI: 25.7 ± 4.7 kg/m^2^) undergoing elective surgery, in accordance with guidelines of the South Birmingham ethics. All tissue was flash frozen prior to RNA extraction.

#### Serum collection from CAD and non CAD subjects

Blood samples were taken prior to surgery and serum levels of a panel of proteins (insulin, adiponectin, leptin, CRP and resistin) were determined and compared to non-diabetic control subjects with no known history of CAD (n = 38, age: 38.0 ± 12.58 yrs; BMI 25.5 ± 2.34 kg/m^2^).

### RNA extraction

RNA was extracted from samples using RNeasy Lipid Tissue kit (Qiagen, UK) according to the manufacturer's instructions. Extraction was followed by a DNase digestion step to remove any contaminating genomic DNA. RNA was quantitated using the Nanodrop ND-1000 Spectrophotometer (LabTech, UK) and 1 μg of RNA from each sample was reverse transcribed using RevertAid™ H Minus M-MuLV Reverse Transcriptase (Fermentas, UK) and random hexamers, according to the manufacturer's instructions.

### Real-time PCR

RT-PCR was performed in a reaction mix containing TaqMan Universal PCR Master Mix (AmpErase UNG), 100–200 nmol TaqMan probe, 900 nmol primers and 25 ng cDNA for all PCR reactions except resistin, where 115 ng cDNA was utilised. All reactions were multiplexed with the housekeeping gene 18S, provided as a pre-optimised control probe (Applera, UK) enabling data to be expressed as delta threshold cycle (ΔCt) values (where ΔCt = Ct of 18s subtracted from Ct of gene of interest). Measurements were carried out on at least three occasions for each sample. Reactions were as follows: 50°C for 2 minutes, 95°C for 10 minutes; then 44 cycles of 95°C for 15 seconds and 60°C for 1 minute. Primer and probe sequences for resistin, adiponectin, PAI-1, tissue plasminogen activator (t-PA) and AGT were as previously described [17,18,23,24]. Leptin and CD45 primers were designed using Primer 3 [25] and their specificity determined using BLAST at NCBI. Primers were designed to amplify across an exon/exon boundary (leptin forward: ACC AAA ACC CTC ATC AAG ACA ATT, leptin reverse: TCC AAA CCG GTG ACT TTC TGT T, leptin probe: ATT TCA CAC ACG CAG TCA GTC TCC TCC A, CD45 forward: CGT AAT GGA AGT GCT GCA ATG T, CD45 reverse: CTG GGA GGC CTA CAC TTG ACA, CD45 probe: ACA ACT AAA AGT GCT CCT CCA AGC CAG GTC T). TNF-α and IL-6 expression were assessed using a pre-designed gene expression assay-on-demand kit (Applera, UK).

### Data analysis

The expression of each gene was compared between depots using the ΔΔCt method [26]. All statistics were performed at the ΔCt stage in order to exclude potential bias due to averaging of data transformed through the equation 2^-ΔΔCt^. Statistical analysis was undertaken using ANOVA or two-tailed student t-test unless otherwise stated (SPSS 12.0 for Windows, SPSS UK Ltd, UK).

### Serum sample analysis

Resistin (R&D Systems, UK), CRP (Life Diagnostics Inc., USA), leptin, insulin and adiponectin (Biogenesis, UK) and) were assessed by use of standard commercial ELISAs all according to the manufacturers' recommended protocols (Resistin intra-assay CV 4.7%, inter-assay CV 8.4%; CRP intra-assay CV 4.4%, inter-assay CV 3.3%; Leptin intra-assay CV 3.7%, inter-assay CV 4.0%; Insulin intra-assay CV 5.96%, inter-assay CV 10.3%; Adiponectin intra-assay CV 3.4%, inter-assay CV 5.7%;). Glucose concentration was determined using the YSI 2300 STAT PLUS according to manufacturer's instructions.

## Results

### Gene expression in epicardial and abdominal depots

**Figure 1:**
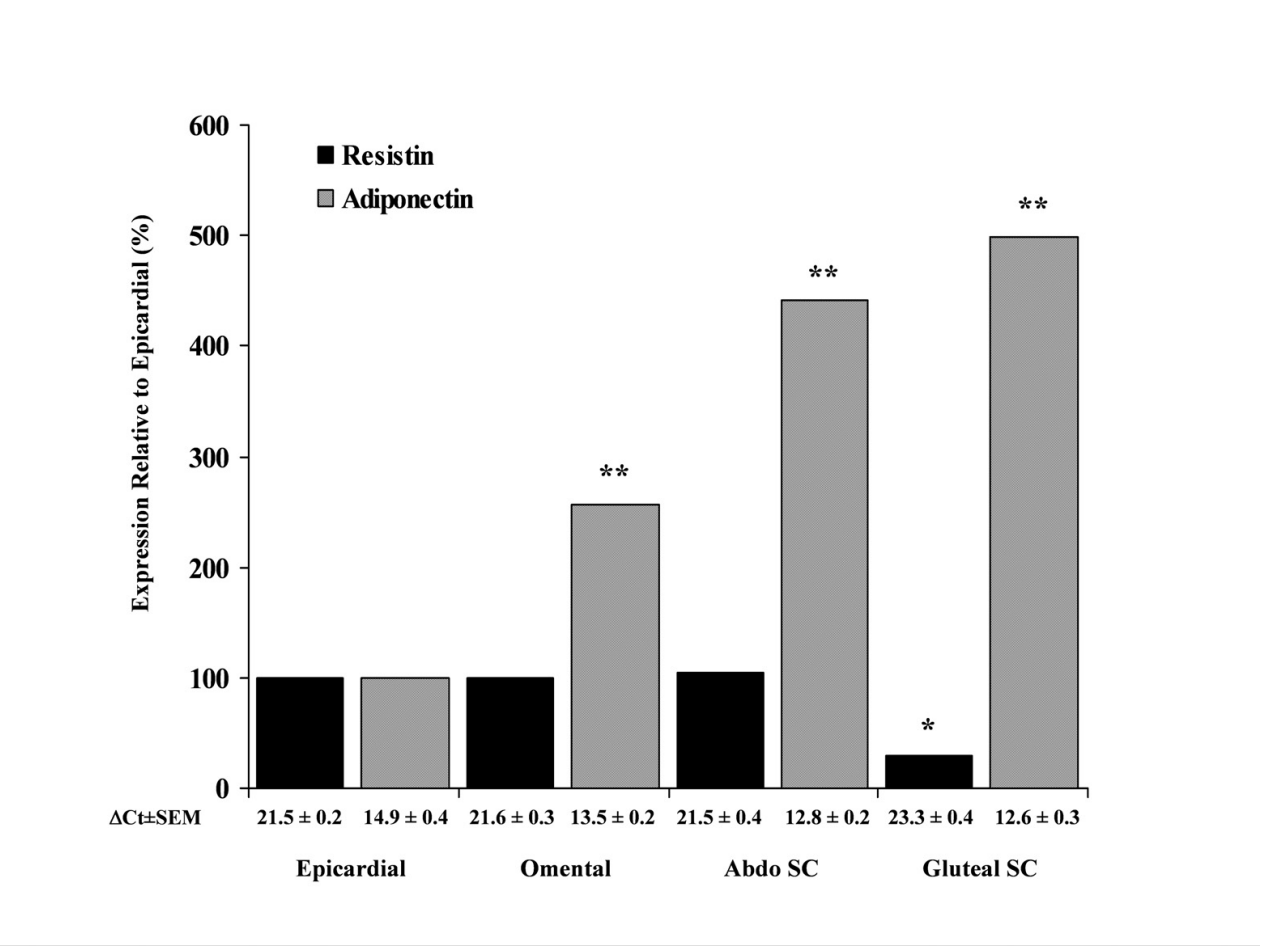
**Expression of Resistin and Adiponectin mRNA**. mRNA levels of resistin and adiponectin mRNA expression are shown relative to epicardial fat (n = 46) which was arbitrarily assigned the value of 100% in abdominal subcutaneous fat (n = 30), omental fat (n = 14) and thigh fat (n = 13) (*P*-values: * *P *< 0.05, ** *P *< 0.01).

Epicardial adipose tissue was assessed for expression of resistin, adiponectin, IL-6, CD45, AGT, TNF-α PAI -1, t-PA and leptin by real time PCR. We determined that the mRNA expression of resistin was 3 fold higher in epicardial adipose tissue than in gluteal adipose tissue (*P *< 0.05), whilst epicardial resistin mRNA expression was comparable with expression observed in the abdominal fat depots (Figure 1). In contrast, adiponectin mRNA expression was 5 fold higher in gluteal adipose tissue than in epicardial adipose tissue (*P *< 0.01). Furthermore, the level of adiponectin mRNA was also significantly lower in the epicardial adipose tissue than in either of the abdominal depots (*P *< 0.01) (Figure 1). IL-6 mRNA expression in epicardial fat was significantly lower when compared with other abdominal depots (Table 2). CD45 was significantly increased in epicardial fat compared with abdominal and thigh depots (thigh vs epicardial *P *< 0.01) (Table 2). The mRNA expression levels of AGT were also increased in epicardial fat when compared with thigh fat (*P *< 0.05), whereas the expression of PAI-1 was significantly lower in the epicardial fat than either abdominal subcutaneous or omental fat. Leptin mRNA expression was significantly lower in both epicardial and omental adipose tissue when compared with both abdominal subcutaneous and gluteal adipose tissue, whilst TNF-α showed no detectable difference across depots (Table 2). In addition sub-group analysis of mRNA of all genes between diabetic (n = 10) vs non-diabetic (n = 36) CABG patients showed no significant differences.

**Table 2:** Comparison of mRNA expression in Epicardial Fat from CAD patients with Fat Depots from non CAD Subjects

	Human CAD Patients	Non-CAD Human Fat Depots					
	Epicardial n = 46	Abdominal SC n = 30		Omental n = 14		Thigh n = 13	

	ΔCt ± SEM	ΔCt ± SEM	% Expression Relative to Epicardial	ΔCt ± SEM	% Expression Relative to Epicardial	ΔCt ± SEM	% Expression Relative to Epicardial

TNF-α	19.74 ± 0.36	19.19 ± 0.50	146	19.46 ± 0.20	121	19.86 ± 0.46	92
AGT	17.19 ± 0.31	18.08 ± 0.32	54	17.10 ± 0.62	106	18.16 ± 0.32	↓51*
PAI-1	19.37 ± 0.41	18.01 ± 0.48	↑257*	17.76 ± 0.54	↑305*	19.76 ± 0.57	76
t-Pa	15.38 ± 0.24	16.38 ± 0.18	↓50*	14.95 ± 0.32	135	16.73 ± 0.34	↓39*
IL-6	23.08 ± 0.83	19.83 ± 0.99	↑951*	18.19 ± 0.86	↑2965**	21.52 ± 0.71	↑295*
Leptin	15.31 ± 0.41	14.07 ± 0.23	↑236*	16.50 ± 0.79	44	13.74 ± 0.21	↑297**
CD 45	18.13 ± 0.46	21.46 ± 0.36	↓10**	20.44 ± 0.27	↓20**	19.79 ± 0.38	↓32**

Expression of mRNA (ΔCt ± SEM) for each gene and depot was analysed. Expression in each depot was compared with that in epicardial adipose tissue from CAD patients which was arbitrarily assigned the value of 100%. Significant increases (↑) and decreases (↓) are highlighted (*P*-values: * *P *< 0.05, ** *P *< 0.01).

### The effect of drug treatment on epicardial gene expression

Treatment with β-Blockers, aspirin, ACE Inhibitors and Ca^2+ ^Channel Blockers did not affect the adipocytokine gene expression in the epicardial adipose tissue (data not shown). However whilst statins were also observed not to affect the majority of adipocytokines the expression of IL-6 was altered. The expression of IL-6 mRNA in those CABG patients receiving statin treatment was reduced to just 16% of the level expressed in those patients not receiving statin treatment (Statin ΔCt = 22.8 ± 0.48(mean ± SEM); Non-Statin ΔCt = 20.1 ± 0.88, *P *= 0.028).

### The effect of GIK infusion on epicardial gene expression

The comparison of gene expression between those CABG patients receiving GIK infusion and those not receiving GIK determined that there was no significant difference in expression between groups across all analysed genes (data not shown).

### Serum sample analysis

The pro-inflammatory cytokine resistin was significantly elevated in the serum of CABG patients when compared to healthy, non-diabetic controls (*P *< 0.01) as were levels of CRP (*P *< 0.001) (Table 3). In contrast, adiponectin was significantly lower in CABG patients when compared to controls (*P *< 0.05) (Table 3). No significant difference was observed in serum leptin levels. Subgroup analysis of diabetic versus non-diabetic CAD patients' serum, identified a significant difference in adiponectin levels (diabetic: 9.8 μg/mL ± 0.9 (mean ± SEM); non-diabetic: 12.7 μg/mL ± 1.0, *P *< 0.05).

**Table 3:** Serum Biochemical Profile

	Control Subjects	CABG Patients	Significance
BMI	25.5 ± 2.34	27.5 ± 3.3	N.S
Fasting Glucose (mmol/L)	5.5 ± 0.79	6.7 ± 0.2	*P *< 0.05
Fasting Insulin (IU/mL)	12.1 ± 0.9	12.1 ± 0.8	N.S
Resistin (ng/mL)	10.53 ± 0.81	16.50 ± 1.69	*P *< 0.01
CRP (μg/mL)	1.28 ± 1.57	9.11 ± 15.7	*P *< 0.001
Adiponectin (μg/mL)	29.1 ± 14.8	11.9 ± 6.0	*P *< 0.05
Leptin (ng/mL)	23.4 ± 4.2	18.5 ± 3.4	N.S

The clinical characteristics are noted between control non-CABG and CABG patient including adipocytokine serum profile, as well as fasting insulin and glucose levels. Values are means ± SEM except BMI (Mean ± SD), N.S represents non-significance.

## Discussion

This study characterised the adipocytokine profile from a unique human epicardial fat cohort comparing this with other adipose tissue depots, evaluating the potential pathogenic paracrine role of epicardial fat on myocardial metabolism. Our study established that metabolic risk markers and pro-inflammatory agents, including resistin, TNF-α and AGT were expressed at similar levels in epicardial fat from CAD patients to those in omental abdominal fat from non-CAD subjects. The impact of the excess mRNA expression of these factors in epicardial fat may represent an important and direct influence on myocardial metabolism due to the intimate association of these tissues. Unlike skeletal muscle there is no fascia separating the myocardium from the adipose layer and both components share the same coronary blood supply [19]. Previous studies have highlighted the paracrine role of ANG II on cardiac function. Perfusion studies with rat hearts have shown ANG II to reduce coronary flow and impair post-ischaemic recovery [27]. As such, regional induction of AGT/ANG II in human epicardial fat may further aggravate myocardial dysfunction. Additionally, data suggest that TNF-α, leptin and other adipocytokines may reduce myocardial contractility implying another possible link between epicardial adipocytokine secretion and cardiac function [5,28,29]. We have also shown significantly lower mRNA expression of adiponectin by the epicardial adipose tissue which supports the previous study showing lower adiponectin protein content in the epicardium of CABG patients when compared with non-CAD subjects [30]. With both anti-inflammatory and anti-atherogenic properties, local secretion of this adipocyte specific molecule may well be important for healthy heart function and as such a reduced level of expression by epicardial fat may represent an important factor in the development of CAD.

We further addressed the potential contribution of macrophages towards the increased inflammatory gene expression profile in epicardial fat compared with other fat depots. Utilising CD45 mRNA expression as a marker for macrophages, we determined that epicardial adipose tissue showed high levels of macrophage infiltration when compared with fat depots from non-CABG subjects. Previous studies have similarly highlighted increased macrophage infiltration in epicardial fat [22]. The high level of macrophage infiltration/activation in this depot indicates that significant local inflammation is occurring. Macrophages are known to express high levels of many inflammatory adipocytokines including resistin [31] and may well account for much of the inflammatory profile of gene expression observed. In this context it is notable that epicardial fat in this study showed low levels of expression of IL-6 mRNA compared with other abdominal fat depots. Whilst reports have implicated IL-6 as a pro-inflammatory cytokine, recent studies have suggested anti-inflammatory properties [32,33]. It is clear that IL-6 levels are raised in the inflammatory state but whether this is as an integral part of the response or in reaction to it remains equivocal. Furthermore, serum levels of both IL-6 and TNF-α are raised in the CAD patients [34] implying that the decrease in IL-6 expression is localised to the epicardial tissue. If, as suggested by Febbraio, Pedersen and others, IL-6 plays an anti-inflammatory role and is beneficial to both glucose uptake and lipid metabolism then the lower levels of mRNA expression in the epicardial adipose tissue of CABG patients may serve only to heighten dysfunction in myocardial fuel metabolism [32,33]. The picture is further complicated by our finding that IL-6 expression is dramatically reduced in those subjects on statin treatment. Previous studies have shown that statin treatment can reduce circulating levels of IL-6 [35], as well as its expression in specific cell types including circulating monocytes [36].

Systemic serum analysis of patients undergoing CABG determined raised resistin and CRP levels, whereas adiponectin levels were decreased, this reduction being exacerbated in CAD patients with diabetes. The anticipated reduction in adiponectin serum levels confirmed previous reports [37], with a negative correlation between serum CRP and adiponectin levels already identified [38]. However this study is the first to report higher serum resistin levels in CVD patients.

In conclusion, this study casts light on the role of human epicardial adipose tissue as a potential paracrine and/or endocrine tissue, specifically within the context of cardiovascular risk. Epicardial adipose tissue shows a similar pattern of expression for a number of key adipocytokines to that of omental adipose tissue. We have also confirmed a high level of macrophage infiltration in this depot which may contribute to the pathogenic gene expression profile in this tissue. Omental adiposity has been shown to be an independent predictor of metabolic risk, with increased resistin, AGT and PAI-1 mRNA expression representing important mediators of inflammatory, fibrinolytic and thrombotic risk [16]. The low levels of adiponectin, which is increasingly becoming associated with the metabolic syndrome and cardiovascular risk [39], strengthens the argument that in CABG patients epicardial adipose tissue represents a negative influence on both cardiovascular outcome and myocardial function. Due to the CAD patients' history the myocardium may have been directly affected by ischemic insult, the gene expression profile that we have described may therefore not be representative of normal epicardial adipose tissue. Further studies are required in order to evaluate the direct effect of these factors on cardiomyocytes and to establish the role of the epicardial fat depot in non-CAD subjects.

## Authors' contributions

AB carried out the serum studies, contributed to the gene expression studies and statistical analysis and drafted the paper. NFS contributed to the statistical analysis. DQ carried out the collection of clinical samples and data. AH contributed to the gene expression studies. DP & RB contributed to the study design and coordinated the patient cohorts and clinical sample collection. SK & PM conceived the study and contributed to the data analysis and drafting of the paper.
